# From classroom to clinic: the role of the clinical learning environment in shaping medical students’ psychological capital

**DOI:** 10.1186/s12909-026-09670-1

**Published:** 2026-06-12

**Authors:** Yinghui You, Zhiyuan Zhang, Ruixin Zhang, Caiyun He, Huiyu Jia, Wenjing Chang, Shan Jiang, Guifeng Ma, Pengtao Liu

**Affiliations:** 1 College of Foreign Languages, Shandong Second Medical University, Weifang, Shandong 261053 China; 2 School of Public Health, Shandong Second Medical University, Weifang, Shandong 261053 China; 3 School of Basic Medical Science, Shandong Second Medical University, Weifang, Shandong 261053 China

**Keywords:** Clinical Learning Environment, Psychological Capital, Medical Education, Psychological Well-being, Structural Equation Modeling

## Abstract

**Background:**

The transition from classroom to clinical practice poses psychological challenges for medical students. This study examines how the clinical learning environment (CLE) shapes psychological capital (PsyCap) among Chinese medical interns.

**Methods:**

A total of 2,589 medical students from 18 universities completed the Dundee Ready Education Environment Measure (DREEM) and Psychological Capital Scale (PCS). Confirmatory factor analysis validated both instruments. Structural equation modeling assessed CLE-PsyCap relationships, controlling for gender and university type.

**Results:**

Both instruments showed excellent psychometric properties (DREEM: α = 0.91, CFI = 0.92; PCS: α = 0.93, CFI = 0.96). Students’ Social Self-Perceptions was the strongest positive predictor of PsyCap (β = 0.33–0.46, all *p* < 0.0001), whereas Students’ Perceptions of Teachers consistently showed negative effects (β = -0.10 to -0.32, all *p* < 0.001). Students’ Academic Self-Perceptions positively predicted resilience (β = 0.19, *p* = 0.0007) but not hope or optimism. Key university students reported higher CLE and PsyCap scores than provincial peers (both *p* < 0.0001). Females perceived more favorable CLE than males (*p* < 0.0001), with no overall PsyCap difference.

**Conclusions:**

The CLE significantly influences medical students’ PsyCap. The adverse impact of teacher perceptions may reflect hierarchical training culture in Chinese medical education. Enhancing social support and learning atmosphere, particularly in under-resourced institutions, is critical for fostering psychological well-being and professional readiness.

## Introduction

Training medical students to become competent healthcare professionals is a core mission of medical education. Central to this endeavor is the transition from classroom learning to immersive clinical practice, where students bridge the gap between theoretical knowledge and real-world patient care [1, 2]. The Clinical Learning Environment (CLE), as the primary context for this transition, plays a critical role in shaping students’ educational experiences, professional development, and psychological well-being [3–5].

Previous studies highlight that effective CLE are characterized by exemplary teaching practices, innovative curricula, and a harmonious social atmosphere [6–8]. These elements promote resilience, self-efficacy and confidence—qualities essential for modern medical practice [9–11]. However, challenges such as high workloads, emotional stress, and inequities in access to resources may negatively impact students’ learning experiences and well-being [12, 13].

In the face of demanding clinical practice, Psychological Capital (PsyCap)—a construct encompassing hope, self-efficacy, resilience, and optimism—emerges as a crucial resource for students to confront challenges effectively. PsyCap not only empowers students to meet the demands of clinical training but also lays the foundation for long-term professional success and well-being. Enhancing PsyCap is therefore essential to prepare future physicians for thriving in an increasingly complex healthcare landscape.

In China, the CLE is shaped by unique sociocultural and institutional factors. Hierarchical medical training, an exam-driven culture, and traditional didactic teaching methods influence how students experience the CLE and develop psychological traits. Systemic disparities between institutions further contribute to varied opportunities for mentorship and skill development [14]. These distinctive characteristics highlight the need to examine how the CLE impacts the development of PsyCap.

Despite growing recognition of the CLE’s significance in medical education, limited research has focused on its nuanced impact on PsyCap in non-western contexts like China. This study seeks to address this gap among Chinese medical students during their internships. By identifying the relationship between CLE and PsyCap, this research aims to provide actionable insights for optimizing clinical training environments. Ultimately, the study aspires to advance the dual goals of clinical competence and psychological well-being, preparing future healthcare professionals to meet the demands of an ever-evolving medical landscape.

This study aims to explore the role of the CLE in shaping medical students’ PsyCap during their internships. By identifying key elements of the CLE that contribute to the cultivation of these psychological traits, this study seeks to provide insights into how the CLE can be optimized to foster not only clinical skills but also the psychological strength necessary for success in the demanding field of medicine.

## Methods

### Participants

Convenience sampling was employed to recruit participants from 18 medical colleges and universities across China, all of whom had practical experience in hospital internships. Prior to participation, informed consent was obtained through electronic signatures. After rigorous data screening, our analysis was based on 2,589 valid responses, representing a response rate of 86.30% out of the total 3,000 responses. The sample demonstrated a comprehensive representation of medical education in China, encompassing students from diverse institutions and academic years. These institutions were strategically chosen from various provinces, including Shandong, Sichuan, Jiangsu, Jilin, Hebei, Liaoning, Beijing, and Shanghai. The average age of the sample was 23.36, with a standard deviation of 9.546. Table 1 shows the demographic characteristics of the sample (Table 1).

**Table 1:** The demographic characteristics of participants (*N* = 2589)

Category	Total *N* = 2589		Key* N = 621		Provincial* N = 1968	
	*n*	%	*n*	%	*n*	%
Gender						
Male	1158	44.7	259	41.7	899	45.7
Female	1431	55.3	362	58.3	1069	54.3
Age						
< 22	594	22.9	156	25.1	438	22.3
22	533	20.6	103	16.6	430	21.9
23	859	33.1	212	34.1	647	32.9
> 23	603	23.3	150	24.2	453	23.0
Major						
Clinical	1805	69.7	426	68.6	1379	70.1
Anesthesia	174	6.7	40	6.4	134	6.8
Nursing	313	12.1	91	14.7	222	11.3
Dental	154	6.0	27	4.4	127	6.5
Other	143	5.5	37	6.0	106	5.4

*Notes, Key: key medical universities; Provincial: provincial medical universities

In the Chinese medical education system, “key medical universities” refer to institutions designated as national priority institutions under the ‘Double First-Class’ initiative or the former “Project 985” and “Project 211” frameworks. These universities receive substantial central government funding, maintain affiliations with top-tier tertiary hospitals, and are recognized for their research output and competitive admission standards. “Provincial medical universities”, in contrast, are regionally administered institutions primarily funded by provincial governments. While they constitute the majority of medical training institutions in China and serve critical regional healthcare needs, they typically have fewer research resources, less prestigious clinical affiliations, and broader admission criteria compared to key universities. This dichotomy reflects a tiered higher education system that shapes resource allocation, faculty quality, and institutional culture.

### Instruments

The study employed a two-part questionnaire. The first section gathered demographic data, including gender, age, and academic major. The second section incorporated scales designed to measure the CLE and PsyCap, systematically derived from core definitions and prior research. By integrating indicators relevant to the medical internship context, the questionnaires for investigating medical students’ perceptions of the clinical learning environment and psychological capital were meticulously developed to align with the study’s aims. Translation and back-translation processes, following Brislin’s (1980) methodology, ensured linguistic and cultural accuracy. A pilot test was implemented among 30 students to assess whether they understood each item in the clinical context. Any item which the students could not understand would be given a validated and clarified Chinese version.

### Measurement of CLE

The Dundee Ready Education Environment Measure (DREEM) was utilized to assess medical students’ perceptions of the CLE (Roff et al., 1997). This tool with 50 statements distributed in five dimensions: (1) Students’ Perceptions of Learning (SPL), 12 items; (2) Students’ Perceptions of Teachers (SPT), 11 items; (3) Students’ Academic Self-Perceptions (SASP), 8 items; (4) Students’ Perceptions of Atmosphere (SPA), 12 items; and (5) Students’ Social Self-Perceptions (SSSP), 7 items. Responses were recorded on a 5-point Likert scale: Strongly agree (5); Agree (4); Uncertain (3); Disagree (2); Strongly disagree (1), with a maximum total score of 250 which indicates the most positive evaluation. The minimum possible score is 50, representing the most negative evaluation; the midpoint of 150 serves as a benchmark, with scores above 150 indicating a more positive than negative perception of the educational environment. Notably, in order to score in consistency, nine of the 50 items (Number 11, 12, 19, 20, 21, 23, 42, 43 and 45) are adapted in reverse coding.

### Assessment of PsyCap 

The 22-items PsyCap scale developed by Siu.(2013) was used to measure medical students’ psychological state in 4 dimensions: Resilience (9 items), optimism (4 items), hope (4 items), self-efficacy (5 items). The sample items include “I can quickly recover from setbacks” (resilience), “I expect more good things to happen to me than bad.” (optimism), “Even when others get disappointed, I know I can find a way to solve the problem.” (hope), “I am confident that I could deal efficiently with unexpected events.” (self-efficacy). All items were rated on a 6-point Likert scale, with higher scores indicating stronger psychological state in the medical internships. These scales have been used in China’s clinical learning environment and were found reliable.

### Reliability and validity assessment

 Prior to SEM analysis, confirmatory factor analysis (CFA) was conducted to validate the factorial structure of both instruments within this Chinese sample. The DREEM five-factor model achieved adequate fit: χ²/df = 2.84, CFI = 0.92, TLI = 0.91, RMSEA = 0.026 (90% CI: 0.024–0.028), SRMR = 0.037. The PCS four-factor model demonstrated strong fit: χ²/df = 2.15, CFI = 0.96, TLI = 0.95, RMSEA = 0.021 (90% CI: 0.018–0.024), SRMR = 0.028. Internal consistency was excellent for both scales: DREEM total α = 0.91, with subscale alphas ranging from 0.78 (SASP) to 0.88 (SPA); PCS total α = 0.93, with subscale alphas of 0.91 (resilience), 0.87 (optimism), 0.89 (hope), and 0.90 (self-efficacy). These metrics confirm that both instruments performed reliably and validly in this Chinese medical student population and justify the subsequent structural equation modeling.

### Data analyses

Data management and analysis were conducted using R (version 4.3.3) and SAS Studio. Descriptive statistics were first employed to summarize the demographic characteristics of the 2,589 valid respondents. Independent-samples t-tests and one-way analysis of variance (ANOVA) were used to examine differences in CLE and PsyCap scores by gender and type of medical university, respectively. The Satterthwaite approximation was applied to account for unequal variances where appropriate. Structural equation modeling (SEM) served as the primary analytical framework to evaluate the complex relationships among CLE dimensions and PsyCap components. Model fit was assessed using multiple indices, including the root mean square residual (RMR), standardized RMR, goodness-of-fit index (GFI), adjusted GFI, parsimonious GFI, and Bentler–Bonett normed fit index (NFI). Model assumptions and overall fit were further evaluated through diagnostic plots, including outlier and leverage diagnostics, Q–Q and P–P plots of residuals, case-level residual distributions, raw residual distributions, and variance-standardized residual distributions. Additionally, visual analyses using histograms, kernel density estimates, and boxplots were performed to compare the distributions of CLE and PsyCap scores across groups, thereby highlighting disparities and central tendencies. To control for potential confounding effects, gender (0 = male, 1 = female) and university type (0 = provincial, 1 = key) were included as exogenous covariates in the SEM, with direct paths to all PsyCap components. Together, these comprehensive statistical approaches enabled a thorough examination of the data and provided robust evidence for the study’s conclusions regarding the influence of the CLE on medical students’ psychological development and well-being.

## Results

### Fit summary of the DREEM

Key fit indices highlighted the robustness of the model: the RMR was 0.0314, and the SRMR was 0.0372, both reflecting minimal discrepancies between observed and predicted covariances. The GFI and Adjusted GFI were 0.9955 and 0.9951, respectively—values nearing the ideal of 1, indicating an excellent fit. Further supporting the model’s validity, the Parsimonious GFI was 0.9427, and the Bentler-Bonett NFI reached 0.9953. Collectively, these indices confirm that the proposed model closely aligns with the empirical data, providing strong evidence of its appropriateness for assessing the educational environment. (Table 2)

**Table 2:** Optimal Model Fit in Educational Environment Assessment

Category	Metric	Value
Modeling Info	Number of Observations	2589
	Number of Variables	50
	Number of Moments	1275
	Number of Parameters	115
	Number of Active Constraints	0
	Baseline Model Function Value	265.4264
	Baseline Model DF	1225
Fit Index	Fit Function	1.2541
	Model DF	1160
	Root Mean Square Residual (RMR)	0.0314
	Standardized RMR (SRMR)	0.0372
	Goodness of Fit Index (GFI)	0.9955
	Adjusted GFI (AGFI)	0.9951
	Parsimonious GFI	0.9427
	Bentler-Bonett NFI	0.9953

### Fit summary of the PCS

The model demonstrated an exceptional fit to the data, as evidenced by a GFI of 0.9985 and an AGFI of 0.9981, both approaching the ideal value of 1. The RMR was 0.0296, and the SRMR was 0.0284, both well within the acceptable range, indicating minimal discrepancies between the observed and predicted data. The Parsimonious GFI was 0.8602, reflecting an efficient parameterization of the model, while the Bentler-Bonett NFI reached 0.9984, confirming the model’s superior fit compared to a baseline model. These findings establish a strong statistical foundation for using the PCS to assess dimensions of psychological well-being. (Table 3)

**Table 3:** Optimal Model Fit in Psychological Capital Scale

Category	Metric	Value
Modeling Info	Number of Observations	2589
	Number of Variables	22
	Number of Moments	253
	Number of Parameters	54
	Number of Active Constraints	0
	Baseline Model Function Value	138.4512
	Baseline Model DF	231
Fit Index	Fit Function	0.2220
	Model DF	199
	Root Mean Square Residual (RMR)	0.0296
	Standardized RMR (SRMR)	0.0284
	Goodness of Fit Index (GFI)	0.9985
	Adjusted GFI (AGFI)	0.9981
	Parsimonious GFI	0.8602
	Bentler-Bonett NFI	0.9984

### Diagnostic plots for SEM 

**Fig. 1:**
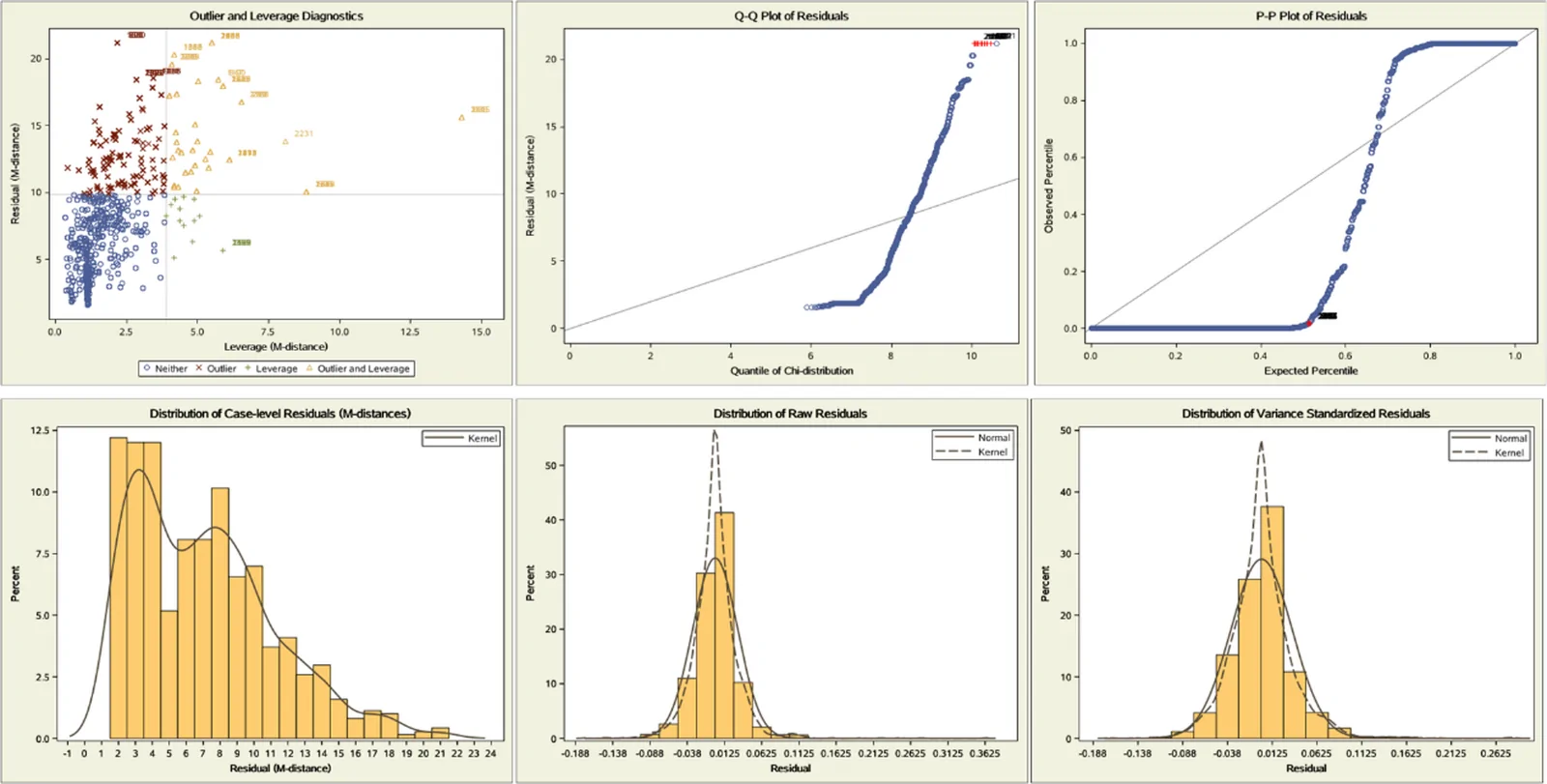
Diagnostic plots for Structural Equation Modeling

SEM were used to assess how the CLE impacts medical students’ PsyCap. Figure 1 presents six diagnostic plots evaluating model assumptions and fit quality: Outlier and Leverage Diagnostics (Top Left): While a few outliers and high-leverage points were identified, the majority of data points fell within the expected range, indicating model robustness. Q-Q Plot of Residuals (Top Middle): Residuals closely aligned with the reference line, particularly in the central region, supporting a normal distribution. P-P Plot of Residuals (Top Right): Residuals followed expected percentiles, further confirming normality and good fit. Case-level Residuals Distribution (M-Distances, Bottom Left): The histogram and kernel density estimate showed a roughly symmetrical, zero-centered distribution, consistent with a well-fitted model. Raw Residuals Distribution (Bottom Middle): Residuals were centered around zero with a bell-shaped curve, closely resembling a normal distribution. Variance Standardized Residuals Distribution (Bottom Right): These residuals also exhibited a symmetrical, normal-like distribution, as expected. Overall, the diagnostic plots confirmed that the residuals were normally distributed, the model was robust against outliers, and the fit was highly satisfactory. (Fig. 1)

### SEM reveals the CLE’s Impact on medical students’ PsyCap

**Fig. 2:**
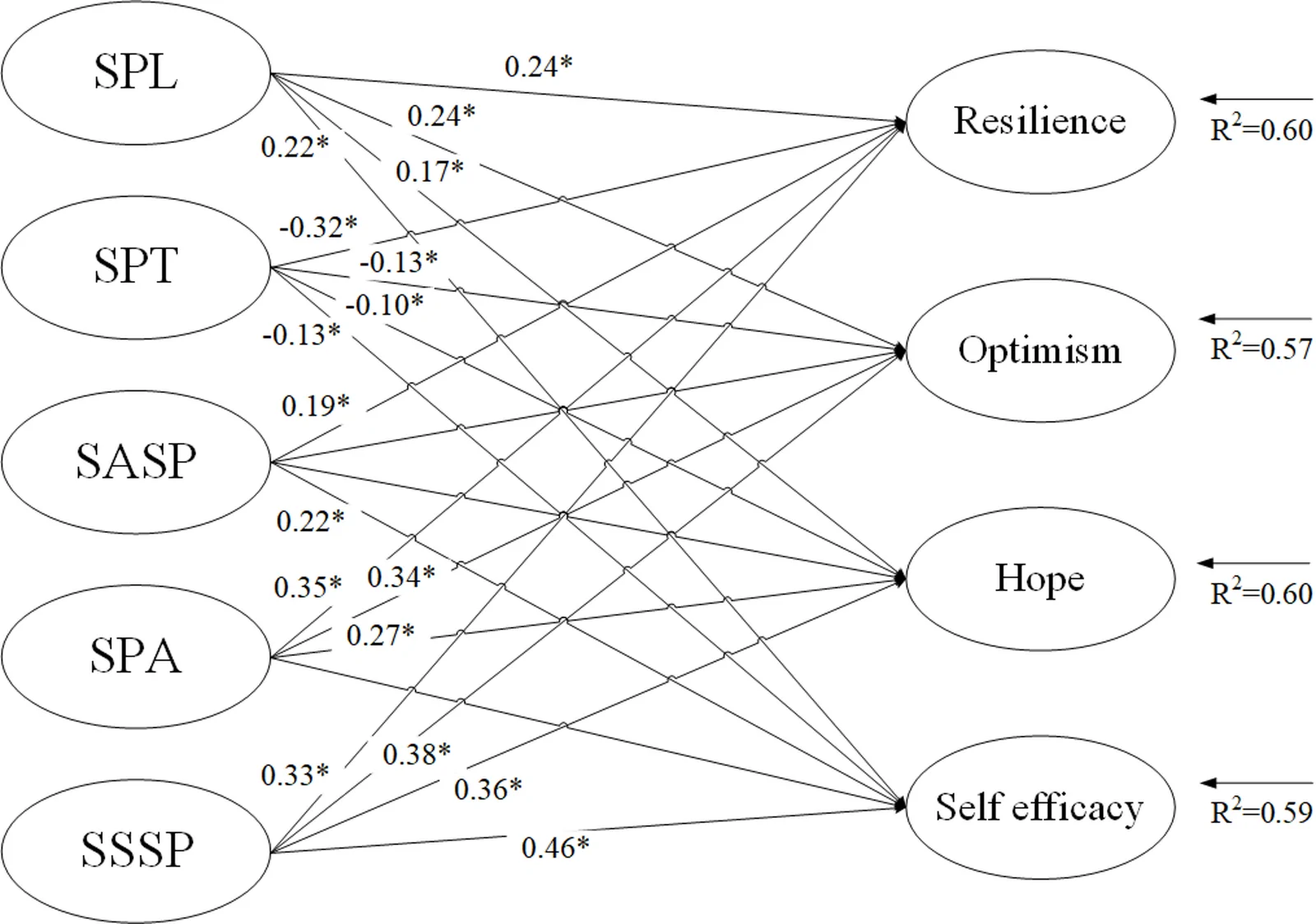
SEM model results showing significant regression paths

The SEM, illustrated in the path diagram (Fig. 2), provided an in-depth analysis of how the clinical learning environment, as measured by the DREEM, impacted medical students’ psychological capital. The structural pathways revealed distinct patterns of influence: SPL exhibited significant positive effects across all psychological traits. It strongly enhanced resilience (β = 0.24, *p* < 0.0001) and optimism (β = 0.24, *p* < 0.0001), with moderate positive impacts on self-efficacy (β = 0.22, *p* < 0.0001) and Hope (β = 0.17, *p* = 0.0005). In contrast, SPT consistently exerted negative influences. It notably reduced resilience (β = -0.32, *p* < 0.0001) and Self-efficacy (β = -0.20, *p* = 0.0006) and demonstrated weaker but still significant negative effects on optimism (β = -0.13, *p* < 0.0001) and hope (β = -0.10, *p* = 0.0001). SASP showed mixed effects. It had a significant positive influence on resilience (β = 0.19, *p* = 0.0007) and a marginal positive trend for self-efficacy (β = 0.22, *p* = 0.064), though its effects on hope (β = 0.09, *p* = 0.4477) and optimism (β = -0.05, *p* = 0.1796) were not significant. SPA emerged as a strong positive predictor, significantly enhancing resilience (β = 0.35, *p* < 0.0001), optimism (β = 0.34, *p* < 0.0001), and hope (β = 0.27, *p* < 0.0001). However, its influence on self-efficacy was minimal and not statistically significant (β = 0.09, *p* = 0.1562). Finally, SSSP was the most impactful predictor across all the dimensions, significantly improving self-efficacy (β = 0.46, *p* < 0.0001), Optimism (β = 0.38, *p* < 0.0001), hope (β = 0.36, *p* < 0.0001), and resilience (β = 0.33, *p* < 0.0001). (Fig. 2)

After controlling for gender and university type, the structural pathways between CLE dimensions and PsyCap components remained robust and largely unchanged in direction and magnitude. Gender showed a significant positive effect on hope (β = 0.08, *p* = 0.003) and optimism (β = 0.09, *p* < 0.001), consistent with the bivariate findings. University type significantly predicted all PsyCap dimensions (β = 0.12–0.18, all *p* < 0.001), confirming the institutional disparities identified in group comparisons. Importantly, the inclusion of covariates did not substantively alter the pattern of CLE-PsyCap relationships, supporting the robustness of our primary findings.

### Gender differences in perceptions of the CLE: insights from statistical and visual analysis

The t-test results revealed a significant gender difference in CLE scores. Female students (*n* = 1431) had a higher mean score of 213.4 compared to males (*n* = 1158) with 207.3, showing a mean difference of 6.09 (95% CI: 3.29–8.90). Both scores exceed the midpoint of 150, indicating an overall positive perception of the clinical learning environment. The t-test results (t = 4.26, *p* < 0.0001) confirmed the significance, supported by a variance analysis (F = 2.08, *p* < 0.0001) validating the use of the Satterthwaite method for unequal variances. These findings underscored a clear gender-based disparity, with females reporting a more favorable perception of the CLE.

**Fig. 3:**
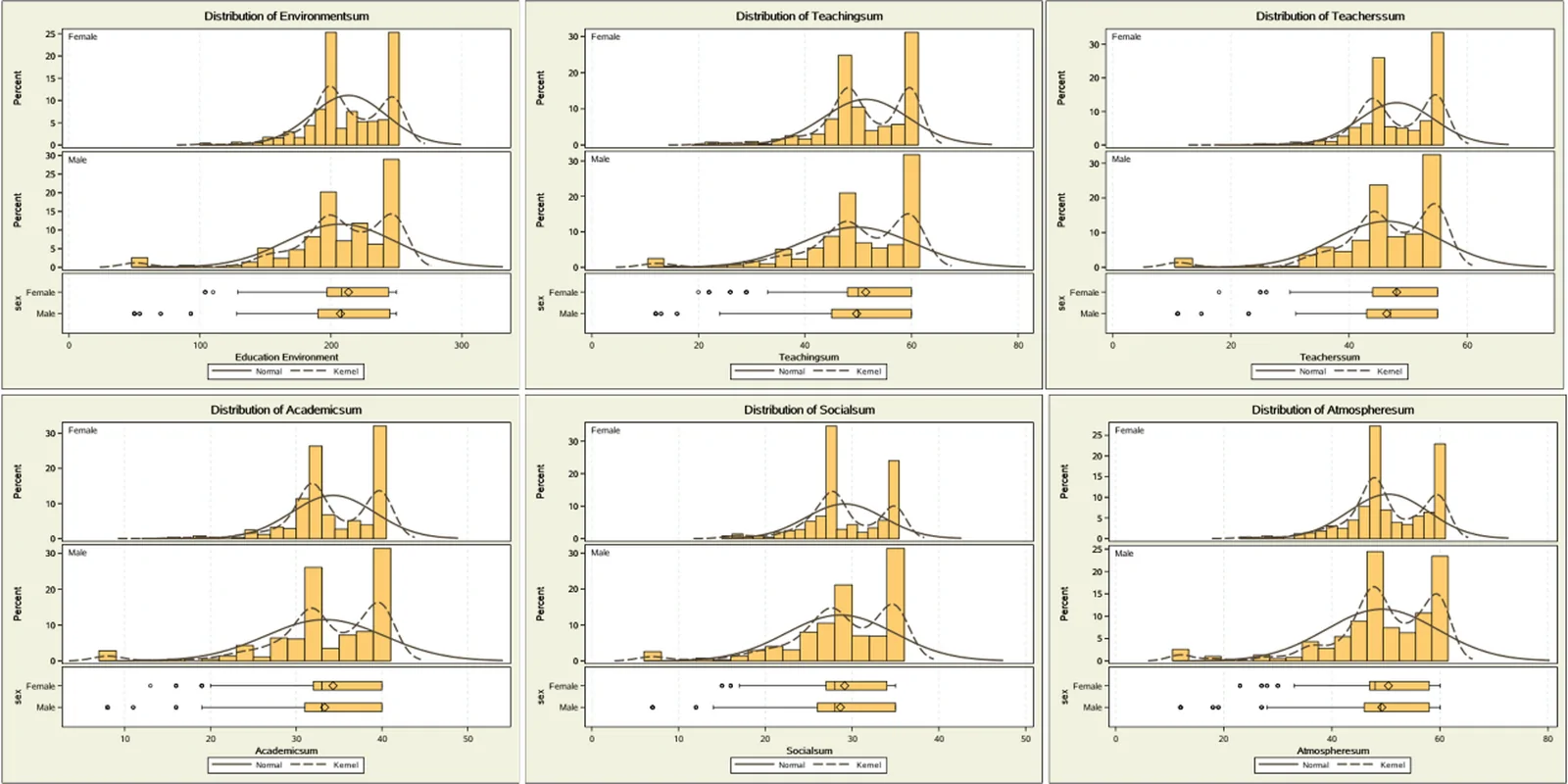
Gender differences in CLE scores between female and male

Figure 3 provided a detailed visual comparison of male and female students’ perceptions across key CLE dimensions, represented by distribution graphs. These dimensions included SPL, SPT, SAP, SSSP, and SPA. The histograms and kernel density estimates revealed a consistent trend: female students outperformed males across all aspects of the CLE. This trend was particularly evident in the higher concentration of female scores in the upper ranges of the distributions. Each graph illustrated that female students’ perceptions of teaching quality, teacher support, academic structure, social environment, and overall atmosphere were significantly more favorable. These findings reinforced the statistical results and emphasized a pronounced gender-based difference in how the educational environment was experienced and evaluated. (Fig. 3)

### Gender differences in PsyCap

**Fig. 4:**
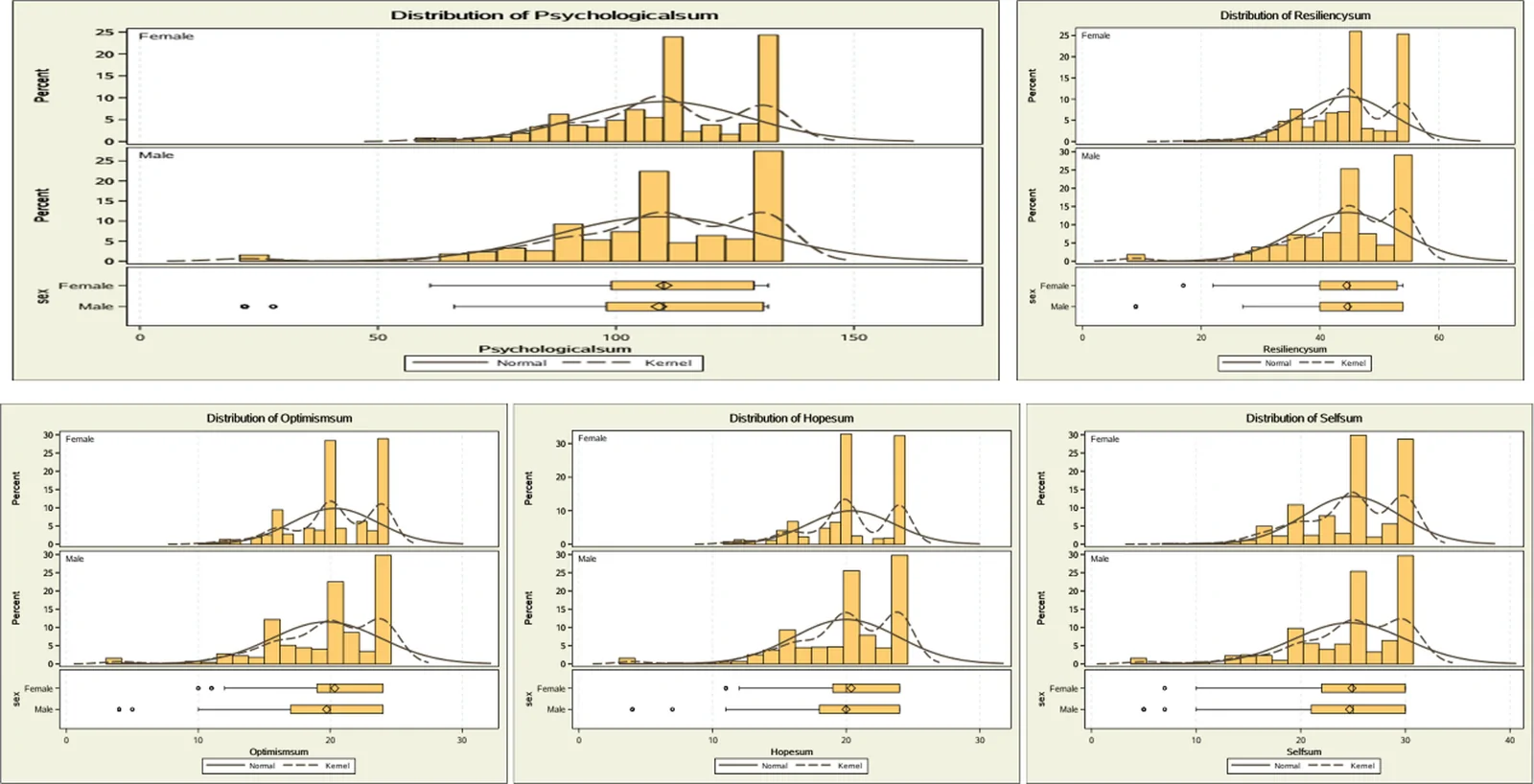
Gender differences in PsyCap scores between female and male

The t-test results indicated that there was no statistically significant difference in the mean PsyCap scores between females and males (t = 1.40, *p* = 0.1606). This suggested that the psychological aspects of the learning environment were perceived similarly by both genders (Fig. 4, Top Left). However, a more nuanced analysis of PsyCap across various dimensions revealed some gender-specific trends: Females showed a slightly higher mean hope score (20.38) compared to males (19.99), with a small difference of 0.39 (*p* = 0.0057). Furthermore, females had a notably higher mean optimism score (20.34) than males (19.72), with a significant difference of 0.62 (*p* < 0.0001). In contrast, there were no significant differences in self-efficacy and resilience scores between male and female participants. This suggested that while certain aspects of PsyCap, such as hope and optimism, might vary slightly between genders, the other core elements of PsyCap—self-efficacy and resilience—were perceived and developed in a comparable manner by both genders (Fig. 4).

### Comparative analysis of CLE and PsyCap: key vs. provincial medical universities 

**Fig. 5:**
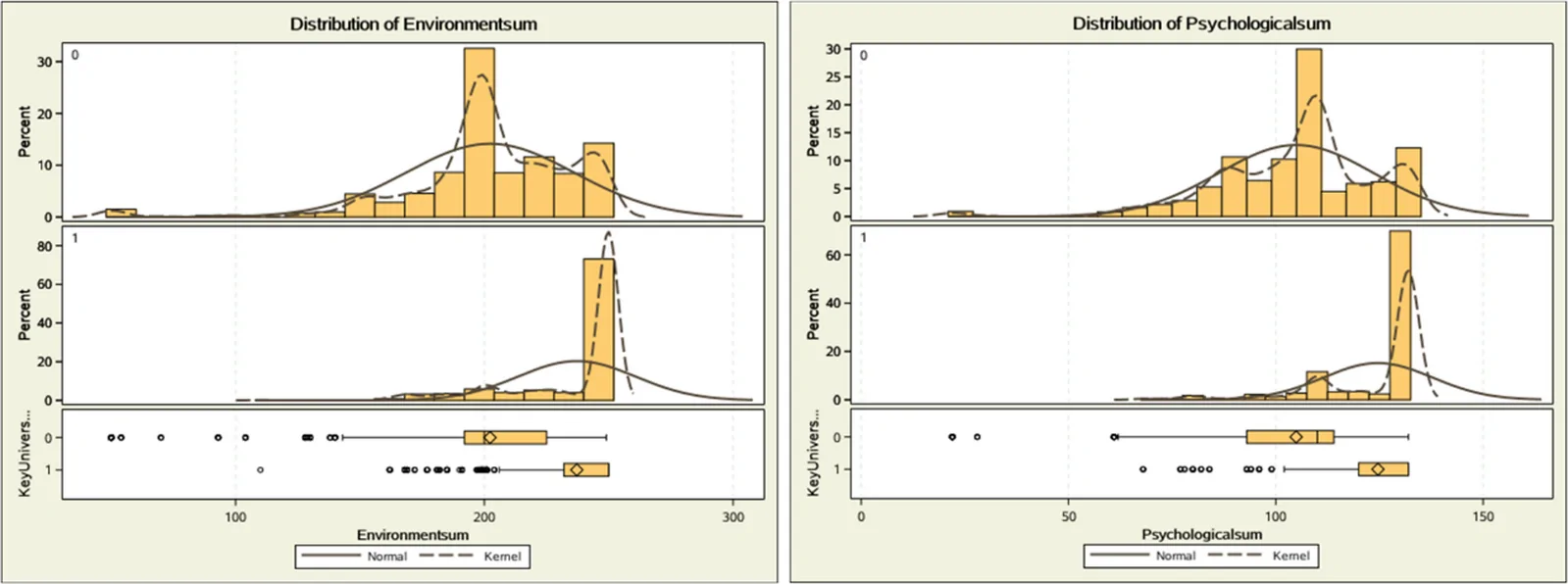
The difference in CLE and PsyCap between key and provincial medical universities

An in-depth comparison of CLE and PsyCap scores between students from Key Medical Universities and Provincial Medical Universities were presented. Using the T-test, the analysis uncovered disparities in mean scores, shedding light on differences in educational and psychological experiences across institutional settings. Figure 5 illustrated the distribution of CLE and PsyCap scores among students from key and provincial medical universities. The histograms paired with kernel density estimates and normal curves revealed a right-skewed distribution for both CLE and PsyCap scores, with a concentration of scores around the median. Notably, the boxplots indicated a higher central tendency for key medical university students in both dimensions, suggesting that they perceived a more favorable learning environment and possessed higher levels of PsyCap. The presence of outliers, particularly in the PsyCap distribution, pointed to a subset of students with significantly divergent experiences. (Fig. 5)

Students at key medical universities reported a notably higher mean CLE score of 237.2 (95% CI: 235.3–239.0), surpassing the 202.3 (95% CI: 200.8–203.8) mean score of their provincial medical university peers. The mean difference of 34.88 (95% CI: 32.02 to 37.74 using the pooled method; 32.49 to 37.26 using the Satterthwaite method) underscored the statistical significance of this gap. The standard deviation figures, 23.58 for key medical universities and 33.85 for provincial medical universities, suggested a more pronounced variability in CLE perceptions among the latter group. (Table 4)

**Table 4:** The difference in CLE scores between key and provincial medical universities

Key	Method	Mean	95% CL Mean		Std Dev	95% CL Std Dev	
No		202.3	200.8	203.8	33.85	32.83	34.95
Yes		237.2	235.3	239.0	23.58	22.33	24.97
Diff (1–2)	Pooled	-34.88	-37.74	-32.02	31.70	30.85	32.58
Diff (1–2)	Satterthwaite	-34.88	-37.26	-32.49			

In the realm of PsyCap, key medical university students again exhibited a superior mean score of 56.10, significantly outpacing the 48.6448 mean score of provincial medical university students. The 95% confidence interval for the mean difference, spanning from − 9.12 to -7.58 using the pooled method and from − 8.10 to -7.71 using the Satterthwaite method, reinforced the substantial difference. The standard deviation figures, 6.36 for key medical universities and 9.09 for provincial medical universities, suggested a more uniform psychological capital experience among students at key medical universities. (Table 5)

**Table 5:** The difference in PsyCap scores between key and provincial medical universities

KeyUniversities	Method	Mean	95% CL Mean		Std Dev	95% CL Std Dev	
0		104.9	104.1	105.7	18.71	18.14	19.31
1		124.6	123.6	125.7	13.14	12.45	13.92
Diff (1–2)	Pooled	-19.72	-21.31	-18.14	17.54	17.07	18.03
Diff (1–2)	Satterthwaite	-19.72	-21.05	-18.40			

## Discussion

This study highlights the pivotal role of the CLE in fostering medical students’ PsyCap during the transition from classroom learning to the clinic practice. Our results offer a robust framework for refining educational strategies that prioritize both psychological well-being and skill acquisition. By employing SEM, we uncover critical insights into the relationships between CLE dimensions and PsyCap components. Positive CLE dimensions, particularly SPL and SSSP, significantly enhance medical students’ PsyCap. However, the negative influence of SPT on resilience, optimism and self-efficacy underscores the need for targeted interventions to address unsupportive teaching behaviors. Notably, students at key medical universities not only rate their CLE more favorably but also exhibit higher levels of PsyCap. This substantial disparity between key and provincial medical universities calls for a reevaluation of institutional policies and practices to ensure equitable educational experiences.

### Key insights from SEM analysis

The SEM results, validated by excellent model fit indices, confirm the theoretical constructs’ practical relevance and underscore the importance of targeted strategies to optimize CLE.

SSSP emerges as the most impactful predictor, exhibiting strong positive effects across all PsyCap dimensions, particularly on self-efficacy and optimism. This underscores the pivotal role of fostering strong social support systems and cultivating a sense of belonging in clinical learning settings [15]. Similarly, SPA also demonstrates substantial positive effects, particularly on resilience, optimism, and hope. These findings emphasize the critical importance of creating a psychologically safe learning environment where students feel valued and supported.

SPL shows moderate positive effects on all PsyCap components, particularly on resilience and self-efficacy which is in line with previous studies [16]. This suggests that engaging and active learning strategies play an essential role in nurturing students’ psychological resources. SASP, while having a modest impact, shows potential in enhancing resilience and self-efficacy, although its influence on optimism and hope is limited. These results indicate that structured and supportive academic experiences contribute to the development of PsyCap, albeit to a lesser extent compared to other dimensions.

Conversely, SPT consistently exerts negative effects on all PsyCap components, with the most significant detriments observed in resilience and self-efficacy. This finding is in agreement with some researchers in their studies [17, 18]. These findings highlight the damaging consequences of unsupportive teaching practices, such as harsh criticism, and lack of encouragement or innovative teaching methods. Addressing these issues requires faculty development programs that focus on promoting empathetic mentorship, constructive feedback, and inclusive teaching practices to counteract the adverse effects of such behaviors [19].

### Alternative explanations for the negative SPT-PsyCap relationship

While the negative influence of SPT on PsyCap is robust, several alternative interpretations warrant consideration. First, the negative association may reflect reverse causality: students with lower PsyCap—particularly those with diminished resilience or self-efficacy—may be more likely to perceive teaching behaviors as unsupportive or critical, rather than the teaching behaviors causing reduced PsyCap. Second, cultural context may shape these perceptions uniquely in China. The hierarchical nature of medical training and the cultural emphasis on respect for authority may lead students to interpret constructive criticism or high expectations as harsh or unsupportive, particularly when feedback is delivered didactically rather than through mentoring relationships. Third, item wording in the DREEM may conflate “perceptions of teachers” with “perceptions of teaching methods”; thus, the negative effect may partly reflect dissatisfaction with traditional, lecture-based didactic approaches rather than interpersonal teacher behaviors per se. Fourth, unmeasured confounding variables—such as student burnout, sleep deprivation during internships, or concurrent examination pressures—may simultaneously erode both PsyCap and SPT scores. Future longitudinal designs are needed to disentangle these potential causal pathways.

### Cultural and hierarchical context of the negative SPT-PsyCap relationship

 The consistently negative influence of SPT on PsyCap warrants deeper contextualization within Chinese medical education culture. As noted in our Introduction, Chinese medical training is characterized by steep hierarchical structures and traditional didactic teaching methods rooted in Confucian educational philosophy, where teacher authority is paramount and questioning is often discouraged. In this context, “perceptions of teachers” may capture students’ experiences of authoritarian rather than facilitative teaching styles—characterized by public criticism, rigid adherence to protocol, and limited psychological safety. Such behaviors may be particularly damaging to PsyCap because they undermine the core psychological needs of autonomy and relatedness identified in self-determination theory. Furthermore, the “hidden curriculum” in Chinese hospitals often normalizes harsh feedback as a rite of passage, which students may internalize as evidence of personal inadequacy rather than constructive guidance. This cultural framing helps explain why SPT emerges as the only consistently negative CLE dimension: unlike atmosphere or social support, which are inherently relational and supportive, teacher-student interactions in this context may be perceived as evaluative and hierarchical, activating threat responses that deplete psychological resources. Faculty development programs must therefore address not only pedagogical skills but also cultural scripts around authority and mentorship to transform SPT from a liability into a resource for PsyCap development.

### Gender-based differences in CLE perceptions and PsyCap

 Significant gender-based differences emerge in both CLE perceptions and PsyCap dimensions, offering critical insights into how male and female students experience and develop within medical education environments. Female students consistently report higher CLE scores across all dimensions, suggesting they perceive the learning environment as more supportive and emotionally attuned to their needs. These findings align with research indicating that women often value collaborative and empathetic interactions, which may enhance their sense of inclusivity and support [20, 21]. In contrast, male students exhibited broader variability in CLE perceptions, potentially reflecting more diverse engagement levels or responses to institutional practices.

Regarding PsyCap, while overall scores show no significant gender differences, female students demonstrate notably higher levels of hope and optimism. This advantage may result from supportive mentorship programs or cultural expectations that encourage adaptive coping mechanisms among women [22]. Conversely, no significant gender differences are observed in self-efficacy or resilience, underscoring shared capabilities in overcoming challenges and recovering from setbacks, essential traits in rigorous medical training.

These findings have practical implications for designing targeted interventions. For male students, fostering hope and optimism through workshops on cognitive reframing and positive psychology can address gaps in these areas. Gender-neutral programs, aimed at enhancing resilience and self-efficacy, such as problem-based learning and stress management training, can provide equitable support. Faculty development programs should also address unconscious biases and ensure inclusive teaching practices, promoting balanced and positive experiences for all students.

### Institutional disparities in CLE and PsyCap

 Institutional disparities in CLE and PsyCap scores between key and provincial medical universities underscore systemic inequities within Chinese medical education. These discrepancies reflect disparities in resources, faculty quality, and institutional culture. Key medical universities often benefit from well-funded infrastructures, access to prestigious affiliated hospitals, and robust academic programs. In contrast, provincial universities frequently face financial constraints, limited access to quality clinical placements, and fewer opportunities for faculty development. Such disparities perpetuate inequities in educational and psychological outcomes.

Key medical university students also exhibit significantly higher PsyCap scores (mean: 124.6) compared to their provincial peers (mean: 104.9), with a mean difference of 19.72 (95% CI: 18.14 to 21.31) and lower variability (standard deviation: 13.14 vs. 18.71). These results highlight the influence of superior institutional support in fostering psychological resilience, hope, and self-efficacy. Students at well-resourced institutions benefit from structured mentorship, access to high-quality learning materials, and a culture of academic excellence, which collectively bolster PsyCap development [23]. Conversely, students at provincial universities face greater variability in CLE experiences, reflected in broader score distributions and outliers, indicative of inconsistent support and learning conditions [24].

The disparities of CLE between key and provincial medical universities point to an urgent need for targeted reforms. Improving faculty training and mentorship programs, particularly at underfunded institutions, is critical. Structured faculty development initiatives can promote teaching practices that emphasize empathy, constructive feedback, and student engagement in clinical practice. Additionally, investments in infrastructure and clinical placements at provincial universities are essential to bridging resource gaps. Leveraging best practices from key institutions, such as integrated learning environments and advanced educational philosophy, could help provincial universities enhance both CLE and PsyCap outcomes.

### Alternative interpretations and limitations of institutional disparities

 While resource-based explanations are compelling, several alternative factors may contribute to the observed gaps between key and provincial universities. Selection effects are plausible: students admitted to key universities undergo highly competitive national examinations (Gaokao) and may possess higher baseline PsyCap, academic self-efficacy, and socioeconomic resources independent of institutional environment. The moderately lower variability in CLE and PsyCap scores at key universities (SD = 23.58 vs. 33.85 for CLE; SD = 13.14 vs. 18.71 for PsyCap) may reflect either homogenization of experience due to standardized protocols or the selective admission of students with more similar baseline characteristics—possibilities that cannot be disentangled in this cross-sectional design.

Response bias could also inflate differences: students at prestigious institutions may report more favorable perceptions due to social desirability or institutional pride, whereas provincial university students might express more critical evaluations reflecting unmet expectations rather than objectively inferior conditions. Furthermore, the geographic concentration of key universities in major cities (Beijing, Shanghai) confounds institutional effects with urban-rural disparities in healthcare infrastructure, patient diversity, and living conditions. We cannot rule out the possibility that observed differences reflect pre-existing student characteristics and geographic advantages rather than purely institutional factors.

These alternative explanations underscore the need for longitudinal, multi-site studies that control for baseline psychological traits—employing propensity score matching or comparable designs—and integrate objective environmental assessments alongside self-reported measures to disentangle selection effects from genuine environmental impacts.

### Limitations and future research

This study is limited by its cross-sectional design, which precludes causal inferences, and its reliance on self-reported measures, which may introduce bias. Future longitudinal studies are needed to explore how CLE impacts PsyCap development over time and to evaluate the long-term effects of educational reforms. Qualitative research could also provide deeper insights into the specific experiences shaping CLE perceptions and PsyCap development.

## Conclusion

The findings underscore the critical importance of optimizing CLE dimensions to enhance PsyCap, a key predictor of medical students’ psychological well-being and professional readiness. Addressing institutional disparities and implementing gender-sensitive interventions are essential to creating equitable and effective medical education environments. By leveraging these insights, policymakers, stakeholders and educators can create a more balanced educational landscape that supports the holistic development of all medical students.

## Data Availability

The datasets generated for the current study are available from the corresponding author upon reasonable request.

## References

[1] Burford B, Whittle V, Vance GH. The relationship between medical student learning opportunities and preparedness for practice: a questionnaire study. BMC Med Educ. 2014;14:223. 25331443 10.1186/1472-6920-14-223 PMC4288662

[2] Illing JC, Morrow GM, Rothwell nee Kergon CR, Burford BC, Baldauf BK, Davies CL, Peile EB, Spencer JA, Johnson N, Allen M, Morrison J. Perceptions of UK medical graduates’ preparedness for practice: a multi-centre qualitative study reflecting the importance of learning on the job. BMC Med Educ. 2013;13:34. 23446055 10.1186/1472-6920-13-34 PMC3599362

[3] Abraha TA, KT WT, Gebre MB, Abrha BA, Haile GB. Opportunities and challenges in clinical learning of midwifery students in public Universities of Tigray Region, Ethiopia, 2020: a qualitative study. BMC Med Educ. 2023;23(1):801. 37884955 10.1186/s12909-023-04765-5 PMC10601281

[4] Nordquist J, Hall J, Caverzagie K, Snell L, Chan MK, Thoma B, Razack S, Philibert I. The clinical learning environment. Med Teach. 2019;41(4):366–72. 30880530 10.1080/0142159X.2019.1566601

[5] Yu W, Yao W, Chen M, Zhu H, Yan J. School climate and academic burnout in medical students: a moderated mediation model of collective self-esteem and psychological capital. BMC Psychol. 2023;11(1):77. 36949548 10.1186/s40359-023-01121-6 PMC10035231

[6] Genn JM. AMEE Medical Education Guide 23 (Part 1): Curriculum, environment, climate, quality and change in medical education-a unifying perspective. Med Teach. 2001;23(4):337–44. 12098379 10.1080/01421590120063330

[7] Sahu PK, Phillips Savage ACN, Sa B. Exploring students’ perceptions of the educational environment in a caribbean veterinary school: A cross-sectional study. J Vet Med Educ. 2020;47(6):668–77. 32053048 10.3138/jvme.2018-0008

[8] Zhao FF. Teaching behaviours of clinical teachers and professional commitment among nursing students: A moderated mediation model of optimism and psychological well-being. Nurse Educ Today. 2023;125:105774. 36921540 10.1016/j.nedt.2023.105774

[9] Khine MS, Fraser BJ, Afari E. Structural relationships between learning environments and students’ non-cognitive outcomes: secondary analysis of PISA data. Learn Environ Res. 2020;23(3):395–412.

[10] Mulrooney A. Development of an instrument to measure the Practice Vocational Training Environment in Ireland. Med Teach. 2005;27(4):338–42. 16024417 10.1080/01421590500150809

[11] Tempski P, Santos IS, Mayer FB, Enns SC, Perotta B, Paro HB, Gannam S, Peleias M, Garcia VL, Baldassin S, Guimaraes KB, Silva NR, da Cruz EM, Tofoli LF, Silveira PS, Martins MA. Relationship among Medical Student Resilience, Educational Environment and Quality of Life. PLoS ONE. 2015;10(6):e0131535. 26121357 10.1371/journal.pone.0131535 PMC4486187

[12] Arora VM, Rosenbluth G, O’Rourke A, Pappas RM, Hamilton AC, Vath RJ, Blanchard AK. Pursuing Excellence: Integrating Clinical Learning Environment Staff and Learners Into the Pursuit of Quality, Safety, Equity, and Value. J Grad Med Educ. 2021;13(2):294–300. 33897969 10.4300/JGME-D-21-00197.1 PMC8054589

[13] Bakhshialiabad H, Bakhshi M, Hassanshahi G. Students’ perceptions of the academic learning environment in seven medical sciences courses based on DREEM. Adv Med Educ Pract. 2015;6:195–203. 25848331 10.2147/AMEP.S60570 PMC4376065

[14] Han J, Yin H, Wang J. A case study of faculty perceptions of teaching support and teaching efficacy in China: characteristics and relationships. High Educ. 2018;76(3):519–36.

[15] Harrison CH, Elmansouri A, Parton W, Myers MA, Hall S, Stephens JR, Seaby EG, Border S. The Efficacy of Frontline Near-Peer Teaching in a Modern Medical Curriculum. Anat Sci Educ. 2018;12(3):236–44. 30332529 10.1002/ase.1827

[16] Behkam S, Tavallaei A, Maghbouli N, Mafinejad MK, Ali JH. Students’ perception of educational environment based on Dundee Ready Education Environment Measure and the role of peer mentoring: a cross-sectional study. BMC Med Educ. 2022; 22(1). 10.1186/s12909-022-03219-8 PMC8925203 35292009

[17] Kohli V, Dhaliwal U. Medical students’ perception of the educational environment in a medical college in India: a cross-sectional study using the Dundee Ready Education Environment questionnaire. J Educational Evaluation Health Professions. 2013; 10. 10.3352/jeehp.2013.10.5 PMC3743137 23967369

[18] Pai PG. Medical Students’ Perception of Their Educational Environment. J Clin Diagn Res. 2014. 10.7860/JCDR/2014/5559.3944 PMC3939516 24596737

[19] Wisener KM, Driessen EW, Cuncic C, Hesse CL, Eva KW. Incentives for clinical teachers: On why their complex influences should lead us to proceed with caution. Med Educ. 2020;55(5):614–24. 33222291 10.1111/medu.14422

[20] Finch J, Waters AM, Farrell LJ. Developing the HERO within: Evaluation of a brief intervention for increasing Psychological Capital (PsyCap) in Australian female students during the final year of school in the first year of COVID-19. J Affect Disord. 2023;324:616–23. 36621678 10.1016/j.jad.2022.12.169 PMC9814284

[21] Kotb AHA, Shazly MM, Mostafa HA. Psychological capital educational program and its effect on nurse interns’ innovative behavior. BMC Nurs. 2024;23(1):544. 39118066 10.1186/s12912-024-02192-5 PMC11311938

[22] Hao J, Wu D, Liu L, Li X, Wu H. Association between Work-Family Conflict and Depressive Symptoms among Chinese Female Nurses: The Mediating and Moderating Role of Psychological Capital. Int J Environ Res Public Health. 2015;12(6):6682–99. 26075725 10.3390/ijerph120606682 PMC4483724

[23] Yang S, Huang H, Qiu T, Tian F, Gu Z, Gao X, Wu H. Psychological Capital Mediates the Association Between Perceived Organizational Support and Work Engagement Among Chinese Doctors. Front Public Health. 2020;8:149. 32528918 10.3389/fpubh.2020.00149 PMC7256625

[24] Wang X, Liu L, Zou F, Hao J, Wu H. Associations of Occupational Stressors, Perceived Organizational Support, and Psychological Capital with Work Engagement among Chinese Female Nurses. Biomed Res Int. 2017. 10.1155/2017/5284628. 10.1155/2017/5284628 PMC5266809 28168198

